# Valorising End-of-Life Mattress Waste into Sustainable Construction Insulation Materials

**DOI:** 10.3390/ma18030647

**Published:** 2025-01-31

**Authors:** Lachlan Thompson, Md Morshed Alam, Fareed Tamaddoni Jahromi, Mostafa Nikzad

**Affiliations:** 1 Department of Chemistry and Biotechnology, Swinburne University of Technology, Melbourne, VIC 3122, Australia; lachlanthompson@swin.edu.au; 2 Department of Civil and Construction Engineering, Swinburne University of Technology, Melbourne, VIC 3122, Australia; mmalam@swin.edu.au; 3 Department of Mechanical Engineering and Product Design Engineering, Swinburne University of Technology, Melbourne, VIC 3122, Australia; ftamaddonijahromi@swin.edu.au

**Keywords:** biomass, cellulose, gels, insulation, materials science, polymer upcycling, waste prevention

## Abstract

Shredded mattress waste was valorised into an insulation material via the addition of a cellulose/urea gel. The addition of the cellulose-based gel was found to successfully bond the miscellaneous shred, creating a composite with a unique pore structure. The composites were tested for their thermal conductivity to explore their use as insulation materials in construction applications. From the testing, the thermal conductivity was found to range between 49 and 60 mW/mK depending on the composition and processing steps. While some of the produced composites showed poor thermal resistance not suitable for an insulation product, we report that additional processing resulted in thermal conductivities that were lower than the existing commercial insulation product (45 mW/mK). Numerical simulations revealed that it is possible to further reduce the thermal conductivity of the samples by optimising the porosity and pore sizes. Hence, there is a strong promise of recycling a common waste product into sustainable building insulation products with further optimisations.

## 1. Introduction

One of the most significant developments in the 20th century was the rise in popularity of plastics and other synthetic polymers. From their initial discovery in the 19th century and use, there has been an explosion of industrial products containing synthetic materials. This popularity has led to the major concern of an ever-increasing problem in the form of plastic waste and pollution [1,2]. The recycling and reuse of end-of-life (EOL) polymer-based materials is an important step for reducing the total waste that is being discarded as landfill and to help further reduce pollution [3].

Recycling has garnered significant commercial interest due to a variety of broad reasons such as economic, environmental, moral and political reasons for the act. Many industries are already implementing their own recycling processing, such as the textiles/clothing industry, in which EOL clothing is collected at many stores to be repurposed. While an individual can take steps to recycle or reuse materials such as donations, upcycling or repurposing, mattresses are an example of a product where these options are difficult to implement, as many charities will not accept mattress donations unless they are in excellent condition due to concerns with cleanliness, biological contamination and poor quality. The contents and bulky size of mattresses also make them difficult to repurpose or make into new products, leaving the only real option in most cases to be disposal, typically via landfill. The recycling of mattresses is also a complex problem, given the broad range of mattress compositions available, meaning that all mattresses on the market will be different from each other and will have different properties within given materials such as different foam densities or different percentages of materials used. Each year in Australia alone, over 1.8 million mattresses are disposed, which is a significant contribution to landfill waste. While there have been significant improvements to mattress recycling within Australia, with approximately 60% of all end-of-life mattresses being taken for recycling, the reclaimed material is still only approximately 40–60% of the mattress, leaving a significant amount of material to be discarded or sent to a landfill, particularly the polymeric content [4].

Mattresses are composed of a variety of materials including polyester, latex, cotton, polyurethane, steel and wood. It is difficult to give a specific breakdown for the composition of a mattress given the variety of brands, styles, supports, standards and product origins. Currently, the only typically recycled material from mattresses is the steel found within spring mattresses, as it can be used as scrap metal, and wood, which can be repurposed into materials such as particleboard. While some of the remaining mixed materials (mainly the foam) can be reclaimed and reused in applications such as carpet underlay, most still wind up as landfill waste.

Recycling of materials can be categorised into four distinct methods: primary recycling consists in the mechanical reprocessing of pre-consumer materials into products with equivalent properties; this is commonly performed with materials such as offcuts or scrap material from manufacturing. Secondary recycling consists in the mechanical reprocessing of used materials into products requiring lower properties, examples of which would include the mechanical shredding of foam materials for use as insulation or the smelting down of metal components to be reused; tertiary recycling consists in the the recovery of feedstock chemical components such as polymer precursors, monomers or additives; and quaternary recycling consists in the use of waste material as a fuel source for producing energy in power production or industrial processing via incineration [5].

Mechanical recycling is one of the most common recycling methods due to its relatively simple processing steps, lower costs and lower waste material. Normally, mechanical recycling is performed as primary or secondary recycling, in which pre-consumer or end-of-life materials can be processed. The mechanical recycling of polymers includes the shredding and breakdown into smaller chips, fibres, pellets or powders, which can then be used in applications such as insulation [6] or as fillers in other formats. In terms of the mechanical recycling of mattresses, the shredding of the polyurethane foams for use as carpet underlay has been reported previously [7]. EOL materials are limited in their use upon mechanical recycling due to potential contamination, both biologically (contact with sources of bacteria, fungi, bugs and human waste) and chemically (dyes, retardants and stabilisers) [8]. The shredding of materials also often has the potential to create microplastics due to the nature of the process if not mitigated, resulting in the generation of finer particulate matter.

A growing concern for the potential environmental impact of polymer disposal and landfilling is the creation of microplastics [9]. Microplastics, as the name suggests, are plastic particles/fibres beneath 5 mm in length, which are typically produced in a variety of ways as a side product during mechanical processing such as shredding, grinding and cutting. Microplastics are also produced when materials are left to degrade, such as when left to be exposed to the elements in a landfill, in which the exposure makes the surface brittle and prone to cracking. Microplastics are of significant concern due to their small size and low density, allowing for their spread via the air, water and soil. Microplastic pollution within the oceans has been a topic of significant importance [10], as the microscopic size of such particles allows for their possible ingestion by aquatic life. This ingestion creates an increasing impact due to bioaccumulation as the smaller lifeforms are consumed by larger ones, posing a further risk of ending up in the human food supply chain [11]. Microplastic production can be reduced in a range of ways, such as improvements to wastewater treatment via methods such as filtration and flocculation, in which the microplastics are collected out of water before it is released into the environment [12]. Currently, approximately 90–98% of microplastics are removed from wastewater via these treatments in the form of sludge; however, there has been shown to be a significant portion discharged into waterways [13].

Further processing of mechanically recycled materials can be performed either with the use of adhesives or moulding techniques. The use of adhesives with mechanically recycled polyurethane is often performed as a way of producing denser foams than what is normally obtained with polyurethane on its own [14]. Mechanically reclaimed polyurethane materials can also be reprocessed without adhesives via methodologies such as compression moulding, in which finely ground particles are subjected to high pressure and temperatures to bind them together. This processing is used within the automotive industry in the primary recycling of waste rigid polyurethane foams being recompressed for other applications within the manufacturing process [15].

An alternative to the primary and secondary recycling of polyurethane is tertiary recycling, in which the foam material is chemically broken down into its components, typically the polyol and isocyanate contents [16]. Thermosetting polyurethane materials work by the crosslinking of PU chains to form a complex network material that cannot be easily broken down thermally. Examples of this include rigid PU foams, adhesives and coatings, which are commonly used industrially for a broad range of applications. The crosslinking mechanism typically works by hydrogen-bonding with the C=O and N-H bonding found in the polyurethane chain, which also allows for the crosslinking of polyurethane [17].

This crosslinking process is a considerable limitation for the chemical recycling process due to the extra complexity and strength of the bonds within the network. This complexity is then furthered, given that the crosslinked polyurethane materials often have further modifications to the network via the addition of filler materials. The filler content/gel material can vary noticeably depending on the purpose, examples of which include plant-derived fibres [18], polyvinyl alcohol [19], polyurea [20], elastomers [21] and isocyanate content, all of which combined severely reduces the viability of larger-scale all-encompassing chemical recycling.

An alternative use case for waste polyurethane foams is the production of rebounded foam materials in which the processed foams are chemically treated using some kind of bonding agent, in which the waste material is bound back together using some form of material. These rebounded materials can be used for a broad range of applications depending on the materials involved. Mortar containing waste PU foams was produced by mixing recycled polyurethane foam with cement-based mixtures [22,23]. Using the blended PU foam in place of the traditional sand in mortar, it was found to be viable as a produce given the low density while retaining strength and some improvements in deformation and shape recovery.

Aerogels containing post-consumer mechanically processed polyurethane were produced by initially mixing clay, PVA, and a PU material and allowing the gel to be freeze-dried [19]. The produced aerogels were found to have excellent mechanical properties, with the modulus increasing with increasing clay/PVA content. It was also found that the addition of a binding agent between the PVA and PU content helped improve the modulus too, leading to a stiffer material.

Another application for rebonded polyurethane foams is sound-absorption insulation, which was produced by mixing the PU foam with an isocyanate-based binder material that was then compression-moulded [24]. It was shown that the increasing thickness and density led to an increase in sound absorption, particularly when adding an air gap between the foam and the wall.

Insulation is a core component within a building, and the primary need for insulation is maintaining the desired temperature within, be it to stay cool in summer or warm in winter. Good insulation is also important as a way of improving the energy conservation of a building, as the use of energy-efficient insulation leads to a reduction in the required energy usage for thermal regulation [25]. Insulation comes in a broad range of styles, such as blow-in (loose fill material blown into a void), foam boards, spray foam, blankets (batts and rolls), structural insulated panels, reflective systems and much more. These insulation materials are also made from a broad range of materials including natural (cellulose, wool) and synthetic fibres (fibreglass), polymers (polystyrene, polyurethane) and inorganic materials such as rock (mineral wool) and concrete.

Rigid PU foams have well-established insulative properties, with the thermal conductivity varying depending on factors such as pore size, gas pressure and composition [26]. More modern approaches for improving the thermal conductivity of rigid foams include the incorporation of materials such as cellulose nanocrystals (CNC) [27], silica aerogels [28] and fly ash [29] into the foam to improve their properties and sustainability.

Cellulose-based aerogels were proposed as an idea for use as a bonding material due to their cost-effectiveness, availability, biodegradability and ease of use. Typically, the process for cellulose aerogels involves the creation of a cellulose-based hydrogel, where the cellulose can be produced from a broad range of naturally sourced materials. The gel can be produced via techniques such as nanofibrilation, in which the cellulose content is sonicated until it forms a gel that is then freeze-dried to form an aerogel [30,31]. Alternatively, cellulose aerogels can also be produced by the crosslinking of cellulose with another agent such as urea [32], isocyanate [33] and citric acid [34].

Aerogels are an excellent choice for the rebonding agent too, given their high insulation performance; one such example is aerogels made from nanofibrilated cellulose, which had dimethylphosphonopropionamide (MDPA) and 1,2,3,4-butanetetracarboxylic acid (BTCA) added within the aerogel networking to improve their flame retardedness. These aerogels were also shown to have an excellent thermal insulation capability, with approximately 33 mW/mK [35].

As cellulose is an abundant and highly renewable material with excellent insulative properties by itself, combining it with mattress foam, another material with established insulative properties, allows for a green and sustainable insulation product via the enhancement and repurposing of a major waste source with poor recycling options, helping to reduce landfilled polymeric waste.

## 2. Materials and Methods

### 2.1. Materials

**Figure 1:**
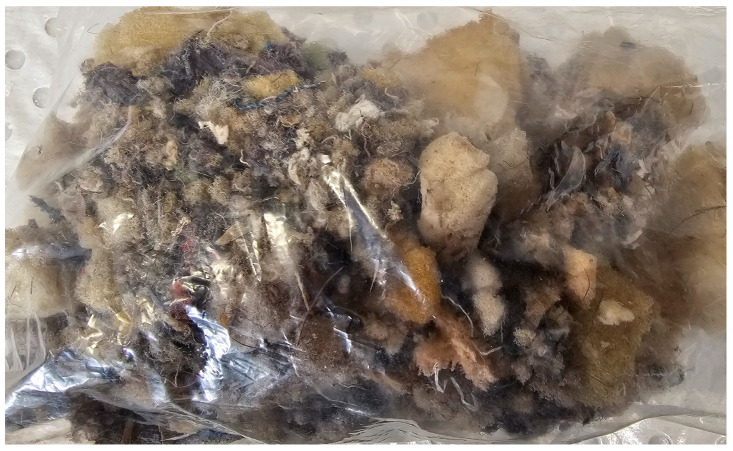
Digital photo showing an example of as-received mattress shred with varying sizes and compositions.

Urea, sodium hydroxide and ethanol (99%) were all acquired from Sigma Aldrich, Melbourne, Australia. Wood pulp was obtained from HRL technologies, Melbourne, Australia, hemp was obtained from the Australian bedding stewardship council (ABSC), Melbourne Australia and cellulose was extracted as per our previous work [36]. Mili Q water was used throughout the experiments. Mattress waste as shown in Figure 1 with various particle sizes and components was received from ABSC from an associated recycling facility and was composed primarily of PU foam, with small amounts of polyester, cotton, polypropylene, nylon and polyvinyl carbonate; any wood or metal components were removed before use.

### 2.2. Cellulose Gel

Cellulose gels were produced by mixing the cellulose and urea in a 1:1 ratio in 60 mL of water until dispersed. Approximately 2 g of NaOH was then added into the solution, and it was mixed for two hours, after which it was placed in a freezer overnight to set. The sample was then washed with ethanol via soaking for two hours to remove excess NaOH and to help further hydrolyse the cellulose/urea gels, after which an additional wash with water was performed. Finally, the samples were frozen to −20 °C and freeze-dried.

**Figure 2:**
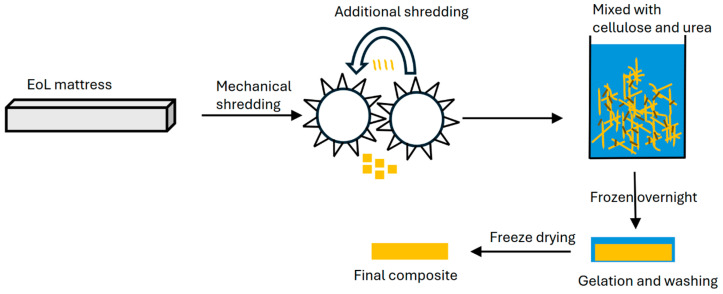
Flow chart of mattress processing to final composite material.

Figure 2 outlines the process for the shred composite samples, where the mattress shred was mixed with the gel at a 1:9 ratio of cellulose:waste in a mould of approximately 120 mm × 120 mm × 20 mm dimensions before the freezing steps.

### 2.3. Material Characterisation

FTIR was performed on a Thermo Scientific Nicolet iS5 FT-IR spectrometer to determine the chemical changes of the produced cellulose/urea gels, with 50 scans at a resolution of 1 being performed per test.

The porosity of materials was determined by cutting samples into approximately 20 mm × 20 mm squares before drying them in an oven overnight at 80 °C and then weighing them (W_i_). The samples were then saturated with water via submersion for 36 h. The samples were removed and cleaned of excess water before being weighed again (W_f_). The % porosity was then determined using the calculation $\frac{W_{f}-W_{i}}{W_{f}}\times 100$.

Optical microscopy was performed to explore the surface morphology of these produced composites. It was performed on an Olympus BX61 microscope with images being taken using multi-focus analysis at various magnifications.

Scanning electron microscopy (SEM) was also performed on the mattress shred composites to explore the surface morphology and how the gel and fibres were combining. It was performed on a Zeiss supra 40VP scanning electron microscope. Before the analysis, the samples were coated with gold via a sputtering coating process.

### 2.4. Thermal Characterisation

To characterise the thermal stability of the produced foam insulation composite, both differential scanning calorimetry (DSC) and thermal gravimetric analysis (TGA) were performed. The testing was conducted on a TA DSC 2920, New Castle, DE, USA (5 °C per minute to 250 °C) and a TA Q50 TGA, New Castle, DE, USA (10 °C per minute to 800 °C), respectively.

**Figure 3:**
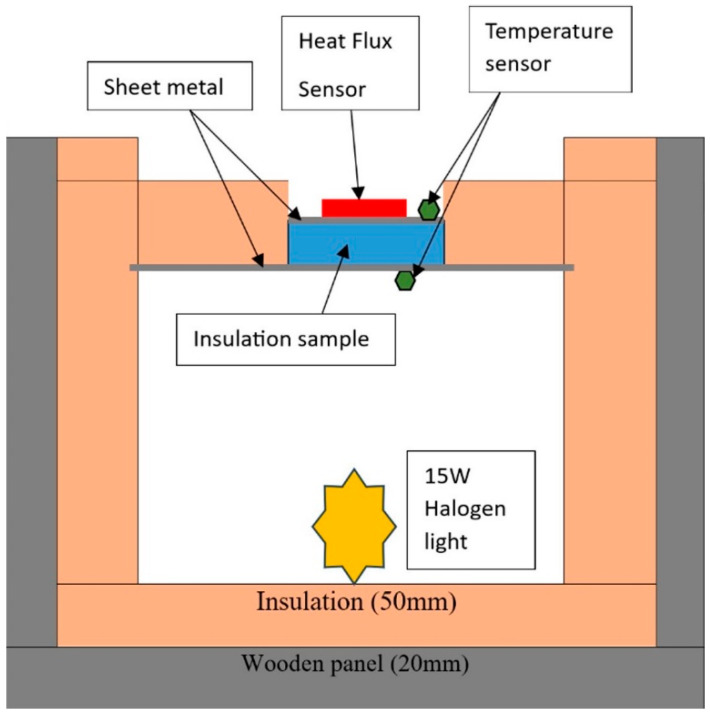
Schematic diagram of the experimental setup.

An experiment was carried out according to the ISO 9869 [37] standard to measure the thermal conductivity of the prepared insulation samples. Figure 3 shows a schematic diagram of the experimental setup. A cube (400 mm × 400 mm × 400 mm) was prepared, where all sides except the top were made of wood and insulated. At the top, a 0.5 mm thick piece of sheet metal was placed. On top of the sheet metal, an insulation panel was placed with a 100 mm × 100 mm square hollow section.

**Figure 4:**
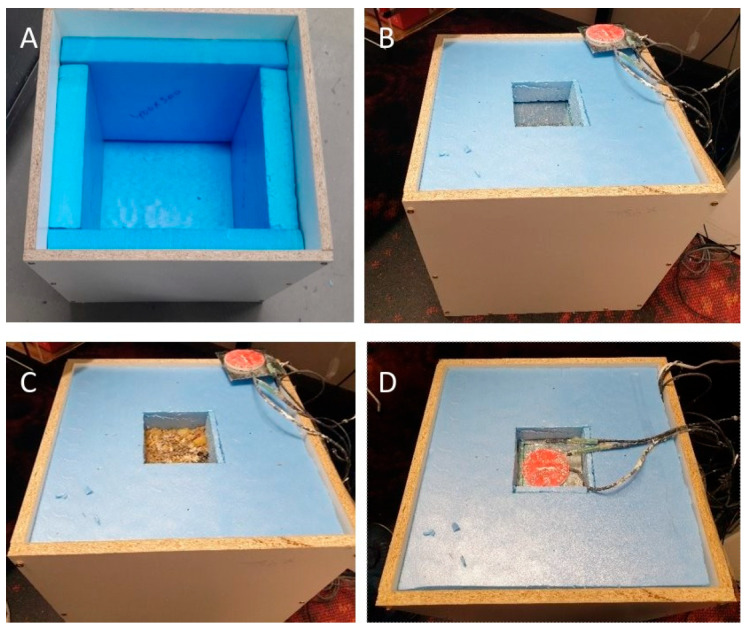
The experimental box with (**A**) insulated on all sides except the top; (**B**) sheet metal and insulation at the top with cut-out square section for insulation; (**C**) insulation sample placed in the cut-out square section; (**D**) sheet metal on top of the insulation panel along with heat flux and temperature sensors.

The sample insulation was placed in that hollow section and covered with another piece of sheet metal as shown in Figure 4. Thermistors, with an accuracy of ±0.05 °C, and heat flux sensors (Hukseflux HFP01, Center Moriches, NY, USA) with an accuracy of ±3% were used to measure the surface temperature on both sides (two on each side) of the insulation and the heat loss through the insulation. To create a temperature difference between the two sides of the insulation, a 15 W halogen light was used inside the box. The box was placed in an ambient temperature of 23 °C. The temperature and heat flux data were recorded through a Voltage Sensor Logger (VSL) at 1 min intervals. Each experiment was performed for at least 24 h to ensure a steady state and constant temperature difference between both sides of the insulation. For each insulation type, three samples were prepared and measured to check the reproducibility of the results.

**Figure 5:**
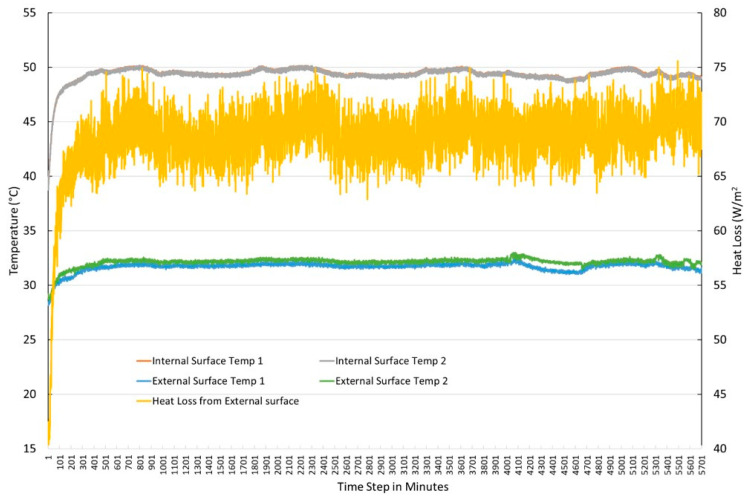
Time series data of surface temperature and heat loss for thermal conductivity measurement experiment.

Figure 5 shows the time series data of the temperature and heat flux of one such experiment. The figure shows that the temperature and heat flux data reached a steady state after approximately 400 min. The temperature differences between both sides were constant at 17.5 °C in this case, which is higher than the minimum requirement of 5 °C for this experimental method. The collected data were then used to calculate the thermal conductivity of the insulation material using the ISO 9869 standard using the following equation:

(1) $$Heatflux\left(\frac{W}{m2}\right)=Thermalconductivity\;(\frac{W}{mK})\times \frac{Temperature\;difference\left(K\right)}{Thickness\;of\;insulation(m)}$$

### 2.5. Numerical Modelling

**Figure 6:**
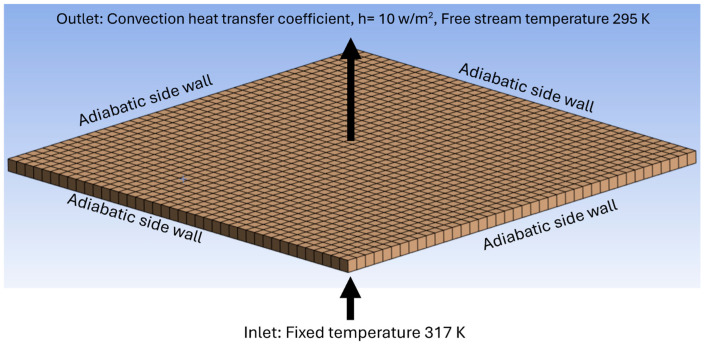
Mesh and boundary conditions of the numerical model.

A numerical model of the developed insulation product was created using ANSYS Fluent 2024 and validated using the experimental data. Figure 6 shows the mesh and boundary conditions used in the numerical model. The mesh size was 0.4 m long, 0.4 m wide and 0.0105 m thick with an element size of 0.001 m. The modelling was carried out using the finite volume approach. The traditional Raynolds-averaged Navier–Stokes equation was solved using the PRESSURE scheme. To solve the turbulence, the K-epsilon turbulence model was used. To model the porous nature of the insulation material, a porous media model was used where an additional source term is used in the standard momentum equation. The source term is composed of two parts: a viscous loss term and an inertial loss term, as shown in the equation below:

(2) $$S=C_{1}v+C_{2}\frac{1}{2}\rho v^{2}$$

where v is the velocity of air in the porous media, ρ is the density of air, $C_{1}$ is the viscous loss coefficient and $C_{2}$ is the inertial loss coefficient. The inertial loss coefficient is not relevant here, as the flow through the porous insulation region is laminar. Hence, it is considered zero. The viscous loss resistance coefficient was set to a higher value of 211,100,000 following Darcy’s law.

For heat transfer through the porous media, a non-equilibrium model was selected where a solid zone that is spatially coincident with the porous fluid zone is defined, and this solid zone only interacts with the fluid with regard to heat transfer. The conservation equations for energy are solved separately for the fluid and solid zones. The impact of the pore size on the heat transfer was investigated via setting two different values of the interfacial area density ratio (IAR). It is the ratio of the area of the fluid–solid interface and the volume of the porous zone. The higher value of 25,434,000 represents the pore size in the order of nanometres, whereas the value of 1000 represents pore sizes in millimetres. Further details of the numerical model input are presented in Table 1.

The thermal properties of air at standard temperature and pressure were considered. The solid-network thermal properties were adopted based on the literature review. This was because the sample mattress waste included a mix of different polymers such as polyethylene and polystyrene and it was impossible to calculate the percentage of each type of polymer and calculate the thermal conductivity. The thermal conductivity of bulk polymers is usually very low, on the order of 0.1–0.5 W/mK, due to the complex morphology of polymer chains [38]. Therefore, in this study, 0.35 W/mK was used, which is within the range. The porosity value of the sample was 0.8, which was determined experimentally in this study.

## 3. Results and Discussion

### 3.1. Cellulose Gel Formation

A cellulose gel was used as the binding agent for the system, as it allows for the use of further waste materials. Cellulose itself can easily be derived from common waste materials such as paper, cardboard and other plant-based materials, with well-established methodologies and pre-existing industrial-scale systems for its manufacturing. Cellulose itself is already commonly used as an insulation product within blow systems, in which loose cellulose material is blown into empty wall cavities or the attic areas above ceilings. Furthermore, a gel that is crosslinked with urea was decided upon, given the low cost and ready availability of urea whilst also having a minimal environmental impact for excess material.

**Figure 7:**
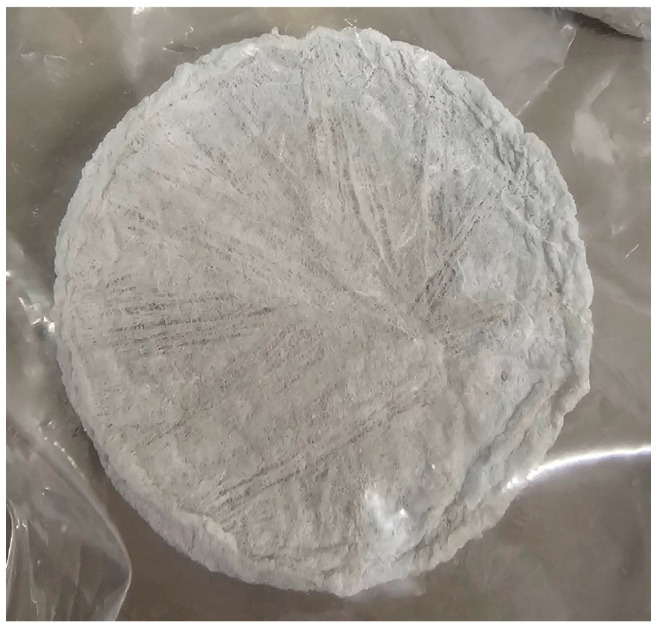
Digital photo of cellulose gel produced by sonication.

A range of different cellulose aerogel production methodologies were explored to determine the best to use as the bonding agent with the waste mattress shred. Initially, a simple methodology using only sodium hydroxide and sonication energy was tested to gel the cellulose solution as shown in Figure 7; however, there were some limitations when attempting to use longer, more fibrous cellulose-based materials in which the fibres would agglomerate and form large, tangled clusters. Scalability was also of concern, as sonication is an energy-intensive process, making it difficult to increase the volumes being treated; a high energy input would also have an impact on the greenness of the developed composite materials. The developed gels also had poor robustness and would commonly crumble upon handling, which further lead to the conclusion that the method would not be ideal for using within a more complex system with the mattress waste.

A gel combining cellulose with urea was then decided upon due to the relatively cheap cost of the material and minimal environmental impact for any waste produced, while also being a simple methodology for the gel formation, only requiring the two to be mixed in a basic solution for a period. The intention was to develop a methodology for a binding agent that would be agnostic towards the source material and not be reliant on bonding with any one polymer, allowing for various mixtures of waste materials to be used.

The cellulose/urea gels were found to be lower in volume in comparison to the sonicated cellulose fibres gels; however, this was deemed unimportant, as the gels were to be blended with another material in the form of the mattress waste, so the system would not be a homogenous aerogel. The urea and cellulose gels were found to have the advantage of being more structurally sound and being able to withstand basic handling and gentle stresses. This can be explained by the improvement in the network via the urea addition, creating a more interlinked system.

**Figure 8:**
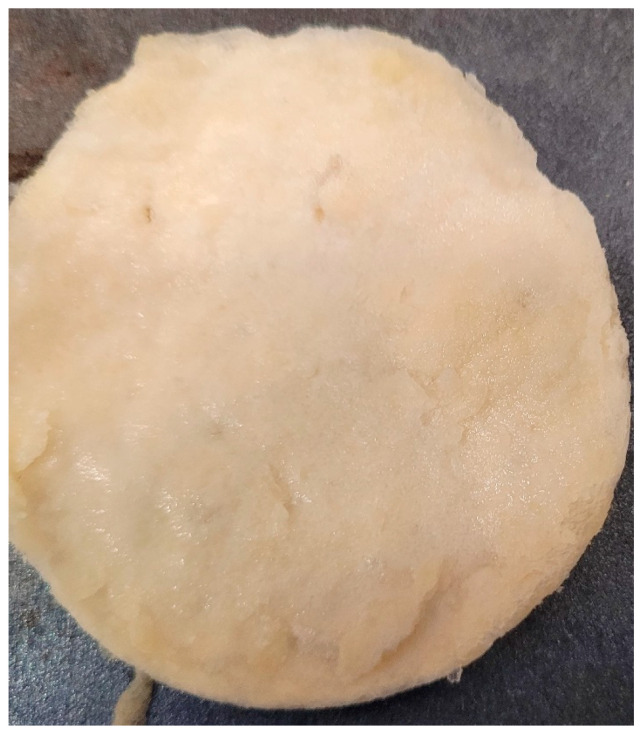
Digital photo of initial cellulose/urea aerogels.

Initially, the methodology for gel involved a significantly higher ratio of urea to cellulose [39] (Figure 8), but over time, it was reduced to being a 1:1 ratio between the two materials, as it was found during the washing steps that there was a significant amount of excess polyurea being removed. Further improvements to the reaction conditions were the reduction of water used, which in turn also allowed for a reduction in the NaOH used, as NaOH facilitates the hydrolysis of cellulose and urea to induce the crosslinking. The time required for gel production was also found to be significantly shorter than what was proposed in the literature. Instead of an overnight mixture, approximately 2 h were found to be adequate for a full gel formation. While the reaction time was potentially even shorter than that, it was decided to remain at two hours to ensure consistency for all testing between samples. These reductions for the methodology all help to improve the sustainability of the insulation material.

As an alternative methodology, foaming agents such as sodium dodecyl sulphate (SDS) were explored for their use in the formation of a cellulose foam; the process was the same with the added steps of mixing SDS after the 2 h mixing process and 4 h in the oven at 60 °C. Ultimately, the benefits from the addition of the foaming agent appeared to be negligible compared to the process without it, while also being an environmental concern at larger quantities such as those required for an industrial scale [40].

**Figure 9:**
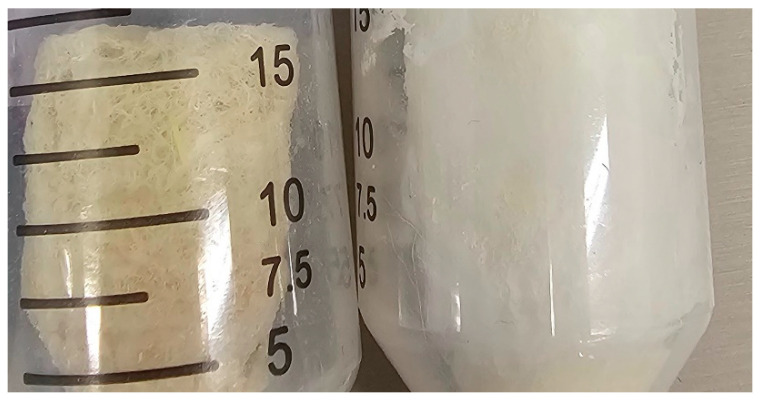
Digital photo comparison of left: hemp-based cellulose gel and right: wood pulp-based cellulose gel.

A comparison between cellulose derived from wood pulp and hemp was explored, since while they are both the same material chemically, the size and aspect ratio between the two are significantly different, with the hemp-derived cellulose (HC) having a larger length per fibre in comparison to the wood pulp-derived cellulose (WC) shown in Figure 9, where the longer length of the hemp cellulose leads to a different structure. The received hemp was also explored for the formation of a gel with urea; however, it was found to be difficult to work with and would agglomerate into a tangled network of hemp fibres instead of forming a gel and failed to work as a binding agent. A hemp gel was successfully produced by increasing the volume of water and mixing for a longer period; however, this methodology was not pursued further, as the chain length impacted the porosity of the final produced gel, which in turn would also impact the insulation properties, as the pore size has a direct impact because the space for heat to transfer readily is reduced, as the pore size reduces the space for free movement.

While the porosity and particle size play an important role within the context of a homogeneous material containing exclusively the crosslinked cellulose fibres, it is unclear how important these factors are when utilising the gels as a bonding agent within another system, as the gel network is now being interconnected with the porous components of the reclaimed mattress waste.

### 3.2. Fourier Transfer Analysis

**Figure 10:**
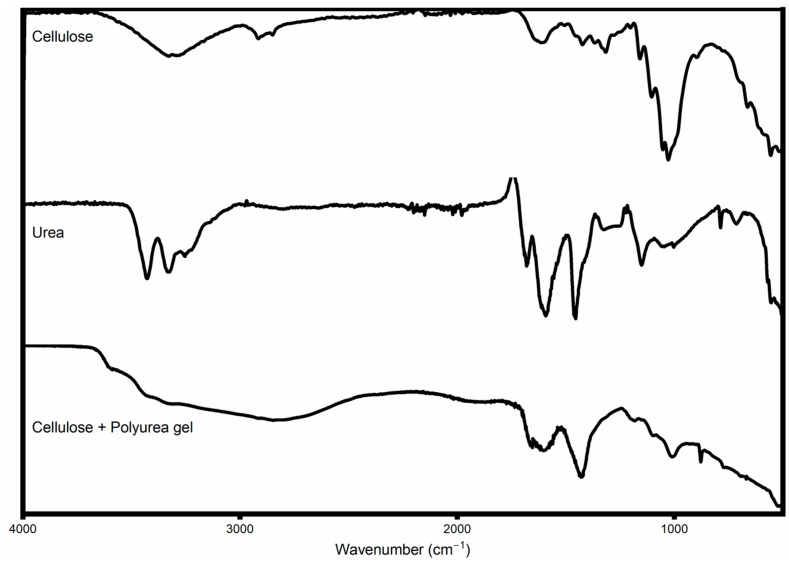
FTIR analysis of urea, cellulose and cellulose and urea gel.

From the FTIR analysis (Figure 10), the most noticeable difference between the original cellulose and the urea/cellulose gel is the broad major peak found formed at approximately 3500 cm^−1^, which can be explained by the addition of NH groups from the urea portion of the composite. There is also a strong reduction in the peak at approximately 1000–1100 cm, which is associated with the C-O-C bonding found within cellulose, which suggests that some bonds have been broken during the crosslinking process. The increase in the peak at approximately 1400 cm can be explained by the inclusion of nitrogen-containing groups from the urea.

### 3.3. Mattress Waste Shred Composites

**Figure 11:**
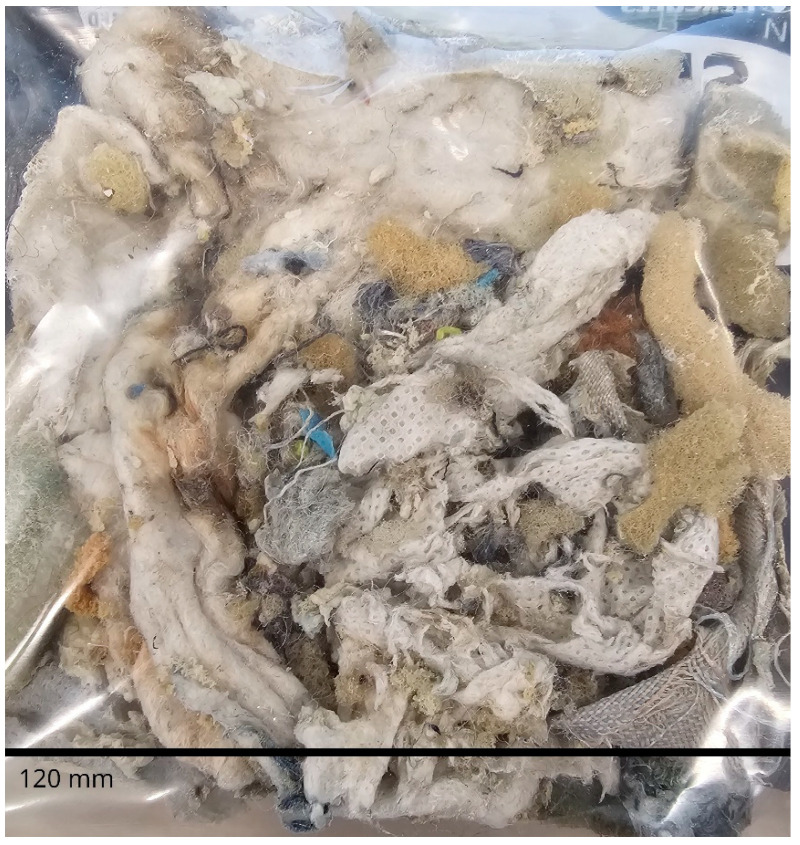
Composite containing random elements of mattress shred.

Using the as-received mattress shred from the recycling plant was performed as a way of using as-is material like that which would be processed within a recycling plant or waste-processing facility without any additional treatment. This was done in the hopes of reducing the environmental impact from microplastics, which would be additionally generated by any further mechanical processing. The other intention of using as-received waste was the reduction in the costs of producing the insulation material at larger scales, as any further processing required incurs additional costs from machinery, maintenance, energy and time. With this, the intention was for the gel and composite process to be agnostic towards the system in which it was being incorporated into. Figure 11 shows a composite produced using a broad range of large pieces of material that is successfully bonded together using the cellulose gel.

A major limitation of the as-received mattress waste materials is the inherent randomness both in size and composition. While the intention for the bonding agent is to be agnostic towards what it is binding together, there are still considerations such as variations in porosity, density and thickness. Most of these variations can be alleviated by further mechanical processing such as shredding, which reduces the general size of individual pieces.

Another limitation that is partly advantageous for mixing the gel and mattress waste is that foams are sponges that absorb moisture into themselves. This makes mixing the shred waste with the gel solution difficult, as the water is removed from the solution. For the mixing, it was explored if it was best to mix the mattress waste with the gel solution during the gelling or after the gel was formed, and ultimately it was found to be best to perform this afterwards, as it required less water and was difficult to perform mechanically at a laboratory scale.

**Figure 12:**
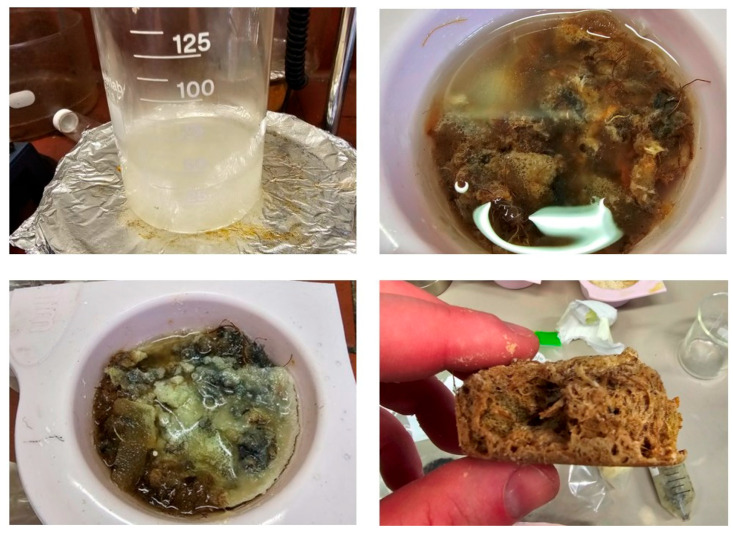
Initial testing for gel–mattress waste composites.

Originally, smaller test samples were produced by combining an excess of the cellulose gel with the mattress waste shred in a silicone mould (Figure 12). From these tests, a rigid material was successfully produced, showing large areas of white solid material on the surface due to excess gel pooling at the bottom of the mould. To reduce this excess material, the ratio of gel to shred was reduced, allowing for less of it to pool.

It was noticed that after several weeks in ambient conditions, some rigidity was lost and the samples became more malleable; this can be explained by the absorption of water from the atmosphere weakening some bonding within the samples. This is unsurprising given the hygroscopic nature of cellulose-based gels. Another explanation for samples being crumbly is due to the various shapes and sizes of components leading to poor adhesion, particularly between larger components.

**Figure 13:**
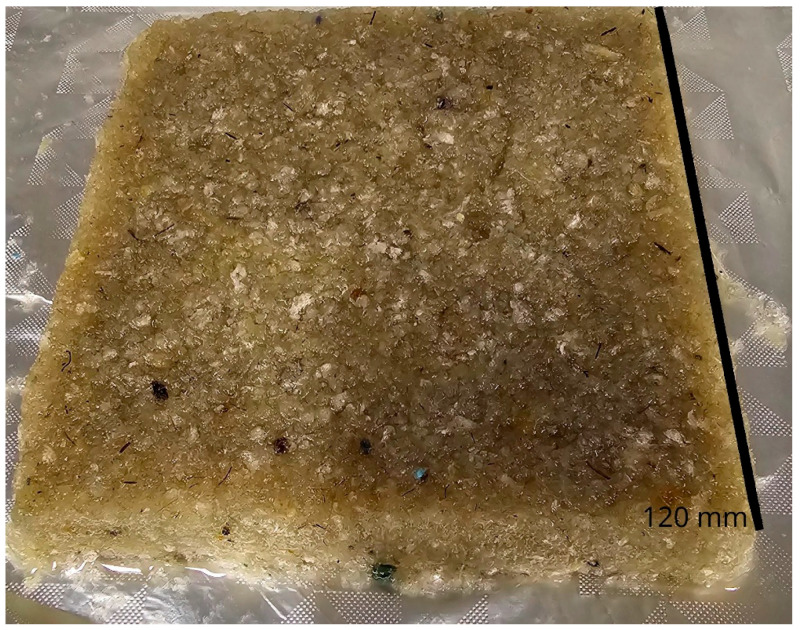
Shredded foam composite during the wash step.

Samples containing mattress-derived waste material that had been further processed via additional shredding were also produced (Figure 13). This additional shredding had the benefit of homogenising the material into a more evenly distributed size, reducing most of the negative traits that the previous samples were exhibiting. It was also noted that this smaller shredded material required less mass to produce samples of equal volume (approximately half the mass). While the additional shredding was initially avoided in the hope that as-received material would be sufficient for use, it became obvious that it was limited in use and reproducibility. This more homogenised distribution was also advantageous due to a reduction in potential air gaps and an increase in surface area, allowing for better binding between individual particles, potentially further helping improve the thermal insulation properties.

The additional shredding also had the benefit of allowing for a one-pot approach to the mixing, in which the foam could be mixed whilst the urea and cellulose were crosslinking. To compensate for the foam’s absorption, however, the water used was increased to approximately 200 mL.

**Figure 14:**
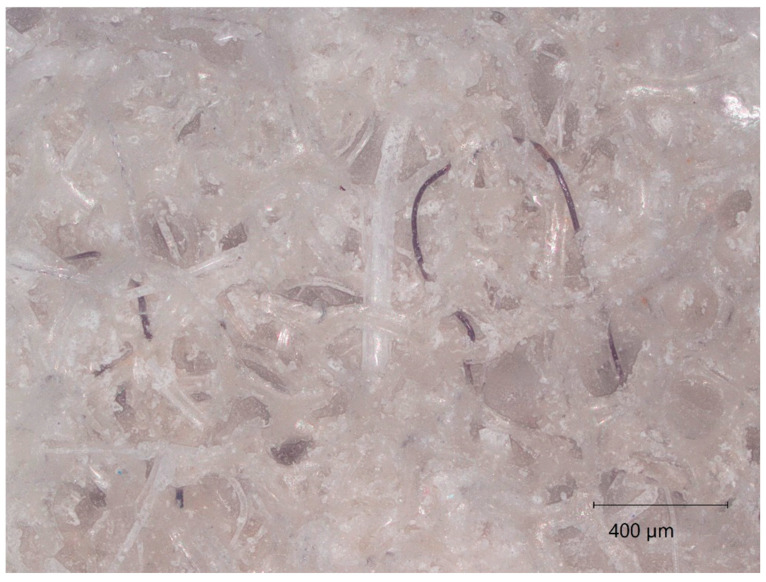
Optical microscope image of shredded foam composite sample.

Figure 14, obtained from optical microscopy, shows how the fibrous network is produced with large interconnecting singular fibres bound together with the cellulose-based gel system. This complex mesh network is believed to be responsible for the improvement of its thermal insulation due to the hindrance of convective heat transfer. It can also be seen that the mechanical treatment has resulted in an improved homogeneity of the composite and the distribution of fibres, allowing for the formation of a randomised distribution of the pore network, which potentially can explain the thermal conductivity of the structure.

**Figure 15:**
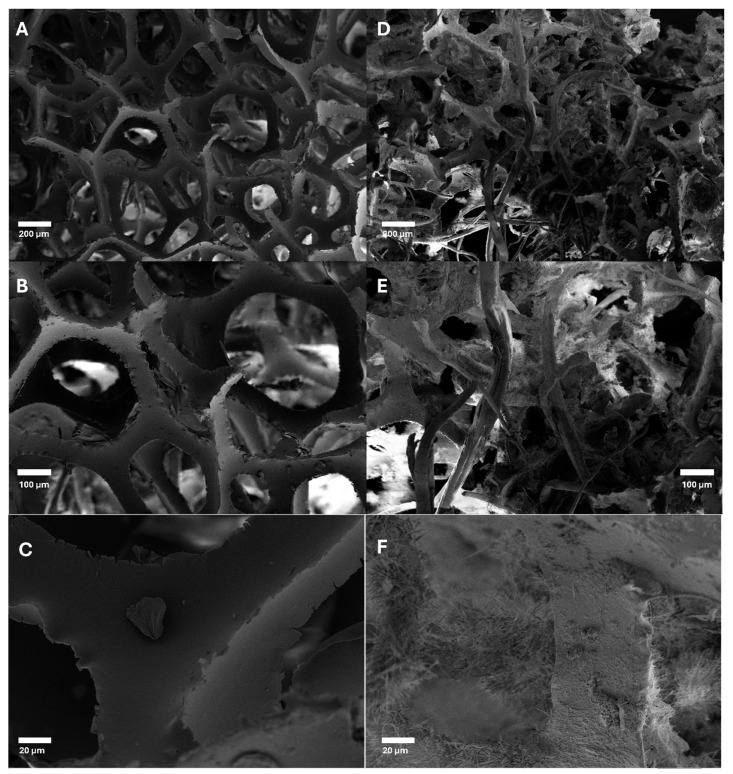
SEM images of as-received foam waste at 50× (**A**), 100× (**B**) and 500× (**C**) magnifications and shredded foam with cellulose gel composite at 50× (**D**), 100× (**E**) and 500× (**F**) magnification.

Figure 15 demonstrates the structural differences between the as-received PU foam waste and the final cellulose/urea + mattress waste post processing. Figure 15A,B show a sample of foam from the as-received foam, where the foam was still highly structured with a consistent porosity, with a pore diameter of 200–300 µm. In the final produced composite foam, these pores have been broken open due to the additional shredding (Figure 15D,E). Another change noticed in the composite foam sample is the addition of fibres interconnecting across the network. This can be explained by both random fibres from the shred mix and excess hemp fibres that have not properly been broken down during the gel formation.

The structure has also become less ordered and more random, creating a complex network of interconnecting fibres and foam parts. This complexity helps explain the thermal insulation properties. The complexity helps hinder the movement of air across the insulation product, in turn reducing the flow of heat.

In Figure 15D–F is also shown the addition of the smaller cellulose fibres on the surface acting as a coating and the altering of the surface morphology compared to the unmodified foam (Figure 15C); these small interlinked fibres are what help bind shredded foam parts together. The interlinked cellulose fibres also help act as a filler between larger pores, furthering the randomness and improving the insulative properties of the produced materials.

### 3.4. Porosity Testing

Porosity was determined using water absorption for the macro-porosity data of the produced samples. From the testing it was found that the composites were on average approximately 80% porous. During the water saturation step, it was also noted that the samples maintained their structure with minimal loss or degradation even during handling, suggesting that there was a robustness to them. This could potentially be explained by the fibrous shred creating a complex interconnected network of bonded material.

### 3.5. Thermal Conductivity Testing

**Figure 16:**
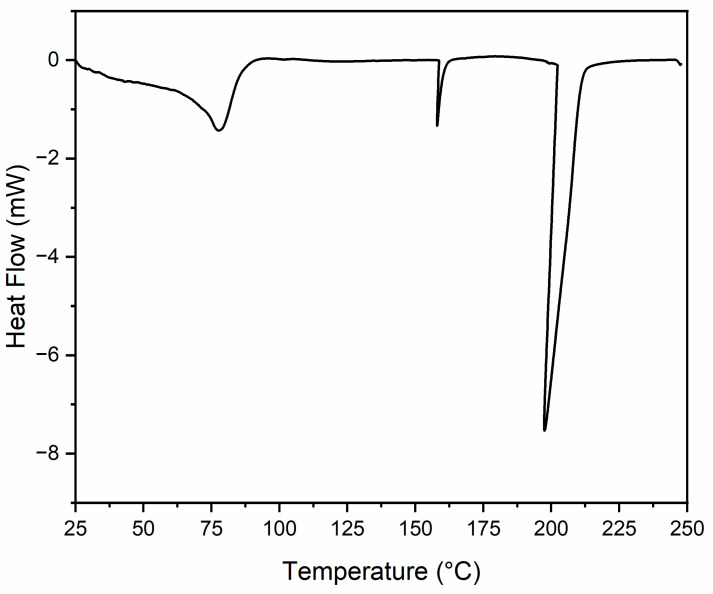
DSC thermogram of cellulose + urea gel.

Figure 16 represents the DSC thermogram for the cellulose + urea gel. The initial peak before 100 °C can be explained by moisture in the gel [41]. The peak occurring at approximately 150 °C can potentially be explained by the urea content degrading, and 180 °C coincides with the thermal breakdown of the cellulose, indicating that the gel has poor thermal stability.

**Figure 17:**
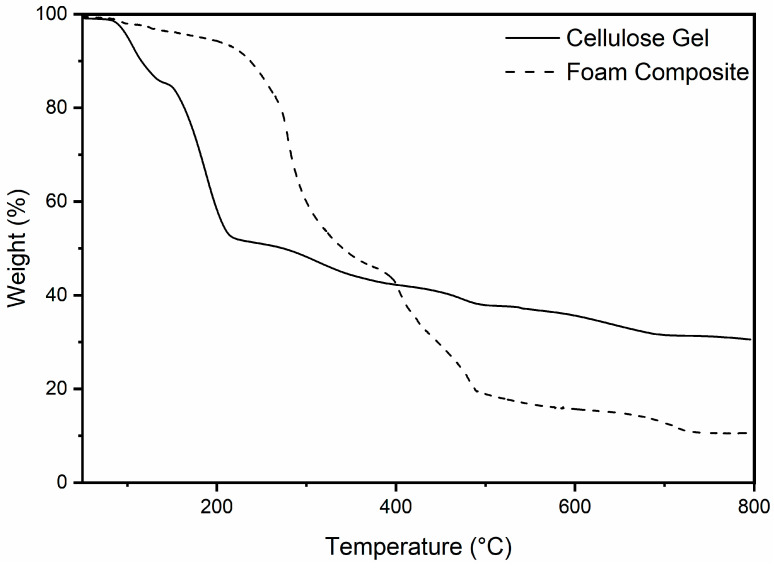
TGA graph comparing the thermal degradation of the produced cellulose + urea gel and foam composite.

Figure 17 shows the degradation of the developed mattress composite, and the cellulose gel used as a binding agent within the system as the temperature increases. The initial loss of mass before 100 can be explained by absorbed water due to hygroscopicity. The mass loss for the cellulose coincides with the result found in DSC, where there is a mass loss at approximately 150 °C and then 180°C. The cellulose gel was shown to continue to reduce to approximately 40% of mass remaining, which can be explained by ash and inorganic content such as sodium from the gel production. Of note is that the thermal stability of the cellulose + urea gel is significantly lower than that found in hemp-derived cellulose (300 °C) [42]. This is potentially due to the gel production weakening the bonds. The foam composite sample was shown to be more thermally stable, with the initial onset being at approximately 250, which coincides with the various polymer materials combusting. This low temperature is of concern, as it shows that the fireproofing capabilities are poor, reducing the viability as a construction material.

**Figure 18:**
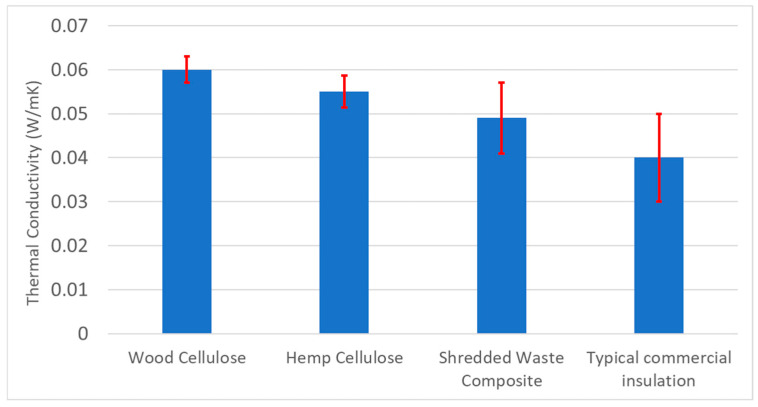
Thermal conductivities of the prepared insulation materials from the as-received mattress shred with wood cellulose (WC) and hemp cellulose (HC) as bonding agents and shredded waste composite (SWC).

Figure 18 presents the thermal conductivity of the measured insulation samples along with their standard deviations. The typical insulation value of existing commercial glasswool insulation batts [43] is also presented for benchmarking purposes. Out of the samples prepared from the as-received mattress shred, the samples prepared with hemp cellulose were found to have better insulative properties (0.055 W/mK) than the wood cellulose (0.060). This can potentially be explained by the hemp cellulose having a longer fibre length, creating a more complex network with the shred during the bonding stages. However, samples prepared out of the as-received shreds have many limitations as described in the previous section. Therefore, further shredding was performed and additional sets of sample insulation materials were developed using the twice-shredded waste composite materials (SWC). Figure 18 shows that the insulation materials developed with further-shredded samples (SWC) had a conductivity 0.049 W/mK, which is the lowest amongst the developed mattress waste insulation and is also closest to the benchmark value of currently available insulation materials in the market. The produced samples of twice-shredded waste composites were thinner (roughly 10 mm thick) and lighter (9 g of foam) in comparison to the previous ones at approximately half the weight and thickness, suggesting an improvement in performance and potentially being viable for the intended insulation use. However, the standard deviation of the twice-shredded waste composite samples is a bit higher than the other two types, which may be due to structural or adhesion issues leading to larger air gaps that lead to an inconsistent performance.

In Australia, the resistance of an external wall insulation should be at least R2.0. In a typical external wall, the air cavity is around 90 mm thick. Hence, to fit the insulation material in that air cavity, the conductivity should be less than or equal to 2/0.09 = 0.045 W/mK. Hence, the thermal conductivities of our samples prepared from further-shredded samples are close to meeting this requirement of an insulation product.

**Figure 19:**
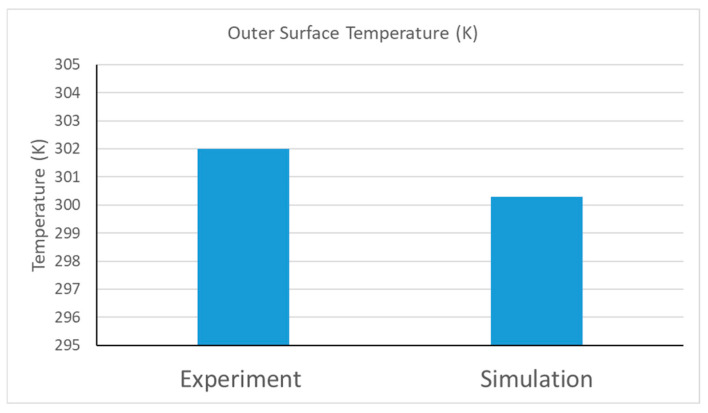
Comparison of experimental and simulation results in terms of outer surface temperature.

The experimental study with further-shredded samples was selected for numerical model development. The parameters shown in Table 1 were used in the numerical model. As it was not possible to calculate the interfacial area ratio density experimentally, it was defined using the trial and error method from the simulation results. The value of the IAR, which provides a close match with experimental data, was considered to be the value of the IAR in the sample. An IAR = 1000 was found to provide excellent validation of the numerical model corresponding to the experimental data, as shown in Figure 19. The outer surface temperature of the prepared insulation sample from the experiment was 302 K, which is a close match with the simulated result of 300.3 K.

**Figure 20:**
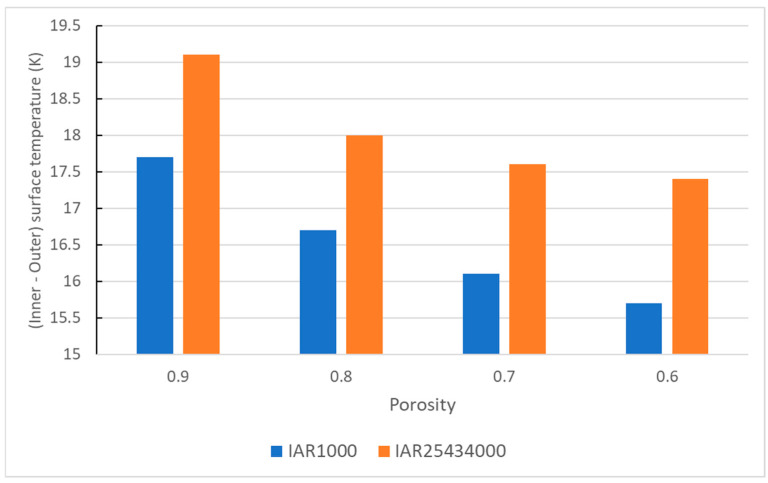
Impact of the porosity and interfacial area density ratio on the heat transfer through a porous medium.

The validated model was then used to explore the impact the of interfacial area density ratio on the heat transfer and outer surface temperature. Figure 20 shows that the heat transfer through the porous medium decreases with an increase in both the porosity and the interfacial area density ratio. However, a change in the interfacial area density ratio has more impact on the heat transfer than the porosity itself. This is because at a higher interfacial area density ratio, the area between the solid and the air is higher and the pore size is lower. When the pore size becomes smaller than the mean free path of air (approx. 70 nm), it restricts the movement of air, and hence the convection heat transfer is greatly reduced [44]. This simulation study is a first step towards simulating the recycled mattress waste to understand the impact of various parameters. In the future, this simulation model will be used to design appropriate insulation materials out of the mattress waste for building applications.

## 4. Conclusions

Cellulose gel crosslinked with urea was successfully used to re-bond shredded mattress waste materials into a singular composite material. This gel-bonding process was found to be agnostic towards the random materials contained within the waste shred, allowing for a robust and complex network to be formed. From testing the thermal properties, it was found that the composite worked well as an insulation material with shredded mattress material, having a thermal conductivity as low as 49 mW/mK on average, which is close to the acceptable value for insulation in buildings. The composites require further optimisations and improvements such as increasing their robustness, improving their fireproofing and investigating their long-term performance for use as an industrially viable insulation material. A numerical model has been developed and validated with the experimental data. The modelling result shows that at a certain porosity, the higher interfacial area density resulted in higher insulative properties of materials compared to a lower interfacial area density ratio. This parameter will be further optimized in a future study. Overall, this study demonstrated that the recycled end-of-life mattress has a significant potential to be used as a building insulation material while mitigating polymer waste in landfills.

## Figures and Tables

**Table 1:** Parameters for modelling.

Parameters	Values
Air thermal conductivity	0.024 W/mK
Solid network thermal conductivity	0.30 W/mK
Air density	1.225 kg/m^3^
Solid network density	800 kg/m^3^
Air-specific heat capacity	1006 J/kgK
Solid network-specific heat capacity	1800 J/kgK
Interfacial area density ratio	1000 25,434,000
Porosity	0.8

## Data Availability

The original contributions presented in this study are included in the article. Further inquiries can be directed to the corresponding author.
